# A Missing Data Imputation Method for Gas Time Series Based on Spatio-Temporal Graph Attention Network—Echo State Network

**DOI:** 10.3390/s26103016

**Published:** 2026-05-11

**Authors:** Jian Yang, Kai Qin, Jinjiao Ye, Yan Zhao, Longyong Shu

**Affiliations:** 1 China Coal Research Institute, Beijing 100013, China; y15703270940@163.com (J.Y.); qinkai@ccri.com.cn (K.Q.); yejinjiao@ccri.com.cn (J.Y.); zhaoyan1016@outlook.com (Y.Z.); 2 State Key Laboratory of Digital Intelligent Technology for Unmanned Coal Mining, Beijing 100013, China

**Keywords:** missing-value imputation, spatio-temporal attention model, ST-GAT-ESN model, Graph Attention Network, Echo State Network

## Abstract

Coal-mine-gas-monitoring data exhibits missing phenomena due to the harsh underground operating environment. Accurate imputation of missing values in gas-monitoring sequences serves as a key data foundation for guaranteeing the continuity of gas data, enhancing the reliability of disaster early warning, and improving the accuracy of mine safety situation analysis and judgment. Aiming at the prevalent random and segmented missing issues in coal-mine-gas-monitoring time-series data, and the limitation that existing imputation methods struggle to accurately capture the nonlinear spatiotemporal correlations and long-range temporal dependencies of such data, this study proposes a missing data imputation method for coal mine gas time-series data based on the Spatio-Temporal Graph Attention Network—Echo State Network (ST-GAT-ESN). Firstly, this method extracts temporal features of the gas concentration sequence using a Gated Recurrent Unit (GRU). Subsequently, it models multiple monitoring points as graph nodes through a Graph Attention Network (GAT), constructs an adjacency matrix based on airflow propagation relationships, and adaptively learns the spatial dependency weights between monitoring points to realize the deep fusion of spatiotemporal features. Finally, it designs a dual-channel Echo State Network (ESN), synchronously inputs the spatiotemporal fusion features of the missing regions before and after, efficiently fits the nonlinear evolutionary trend of the data by virtue of the echo state property of the reservoir, and solves the output layer weights through ridge regression to achieve accurate imputation of missing values. Experimental results demonstrate that, compared with the single-ST-GAT-ESN, ESN, and ARIMA models, the proposed method achieves the optimal imputation performance in both random and segmented missing scenarios within the missing rate range of 5–50%. The three evaluation metrics—Mean Absolute Error (MAE), Root Mean Square Error (RMSE), and Mean Absolute Percentage Error (MAPE)—are reduced by 30–80% compared with the benchmark models. Moreover, the imputation curve achieves the best fitting performance with the ground-truth curve at a 50% segmented missing rate. This study confirms that the ST-GAT-ESN model effectively enhances the adaptability and robustness to complex missing patterns via spatiotemporal collaborative modeling and a dual-channel fusion mechanism, providing a high-precision and highly stable technical solution for ensuring the integrity of coal-mine-gas-monitoring data, and also provides theoretical references and engineering insights for the missing-value processing of industrial time-series monitoring data.

## 1. Introduction

As the main energy source in China, the safe and efficient mining of coal is crucial to national energy security. However, gas disasters remain one of the primary factors restricting the safe production of coal, and incidents such as gas overrun and gas outburst occur frequently [1]. With the advancement of intelligent coal mine construction, gas-monitoring systems based on sensor networks have become the primary means of ensuring underground safety. However, in the actual complex underground environment, affected by various factors, including electromagnetic interference, sensor malfunctions, network transmission interruptions, and equipment calibration and maintenance, gas-monitoring data often suffers from different degrees of loss or interruption [2]. Such data discontinuity seriously impairs the integrity of time-series information, leading to deviations in subsequent data mining, trend prediction, and gas disaster early warning models, and even triggering false alarms or missed alarms, which poses great potential risks to coal mine safety production. Therefore, developing a high-precision imputation method for historical gas time-series data under high missing rates and complex working conditions, and recovering the spatiotemporal characteristics of the data, is an urgent requirement for improving the gas disaster early warning capability of coal mines.

In the research on the missing-value imputation of time-series data, the coal mine field is an important application scenario, mainly focusing on the integrity of safety parameters and production data. In the early warning system for coal mine gas outburst [3], researchers implemented linear regression imputation, K-Nearest Neighbors (KNN) imputation, and matrix factorization imputation on a big data platform, and relevant optimization methods were proposed in [4], which used time-series models to fill in missing values of coal mine production data to ensure the accuracy of subsequent prediction results. Subsequently, researchers mainly adopted multiple imputation technology based on random forests to address the issue of missing data. Aiming at the missing coal mine ventilation parameters, researchers [5] proposed a multiple imputation method based on Multivariate Imputation by Chained Equations with Random Forest (MICE-Forest). This method uses non-missing ventilation parameter data to predict and fill in missing values through an iterative approach. Studies have shown that this model can effectively maintain the mean convergence of the imputed data, and still maintain high imputation accuracy under the condition of a high missing rate. Common imputation methods [6,7] include regression prediction, propensity score method, and Markov Chain Monte Carlo method. These methods analyze the distribution characteristics of known data and generate multiple possible values for filling, thereby improving the accuracy and reliability of imputation results. By comparing machine learning and statistical learning [8], it is pointed out that machine learning models can better capture the nonlinear relationships in the data, thereby improving the imputation accuracy. In recent years, deep learning methods represented by Recurrent Neural Networks (RNNs) and their various Long Short-Term Memory (LSTM) networks have achieved remarkable results in time-series prediction and imputation, which can capture long-range temporal dependencies. In other application fields. Qi Jiandong et al. [9] developed a hybrid model combining Time Series Information Transformer and Patch Transformer for Time Series (TSIT–PatchTST), which improved the imputation accuracy of net ecosystem exchange for long, continuous, missing-data scenarios. Zhan Zhaokang et al. [10] proposed the use of a multivariate spatiotemporal fusion network to extract potential information about missing data, which effectively improved the imputation accuracy of missing fan data compared with traditional methods. Su Jia et al. [11] proposed a conditional generative adversarial imputation network, which significantly reduced the mean square error for large sample sizes.

The aforementioned studies have all achieved certain results in improving the accuracy of missing-value imputation, but there are still obvious limitations in the scenario of coal mine gas time-series data. On one hand, traditional methods such as linear regression and KNN [12,13,14,15,16,17,18] are difficult to capture the nonlinear spatiotemporal correlations between gas concentration and multiple monitoring points, and are prone to trend deviation under high segmented missing rates; on the other hand, ensemble learning methods such as random forest have insufficient ability to model long-range temporal dependencies and cannot adapt to the dynamic evolution characteristics of coal-mine-monitoring data; although deep learning methods such as LSTM [19] have attempted to fuse spatiotemporal features, the single recurrent network structure is prone to the gradient vanishing problem, making it difficult to simultaneously mine global spatiotemporal correlations and accurately fit the contextual information of missing regions. In addition, most existing methods focus on a single missing pattern and exhibit poor adaptability to the commonly occurring random and segmented mixed missing scenarios in coal mine sites. To solve the above problems, this study proposes a gas time-series missing data imputation method based on ST-GAT-ESN. Firstly, this method extracts the temporal dependency features of gas concentration at a single monitoring point using a Gated Recurrent Unit (GRU) [20], then uses a Graph Attention Network (GAT) [21,22,23] to adaptively mine the spatial propagation correlations among multiple monitoring points to realize the deep fusion of spatiotemporal features; subsequently, it constructs a dual-channel Echo State Network (ESN) [24], synchronously inputs the spatiotemporal features of the missing regions before and after, efficiently fits the nonlinear temporal trend by virtue of the echo state property of the reservoir, and obtains accurate imputation results by calculating the output layer weights through ridge regression. Finally, we simulate the segmented and random missing scenarios under different missing rates, and conduct multi-dimensional comparisons with three other models from the perspectives of Mean Absolute Error (MAE), Root Mean Squared Error (RMSE), and Mean Absolute Percentage Error (MAPE).

## 2. Materials and Methods

### 2.1. Model Architecture

**Figure 1:**
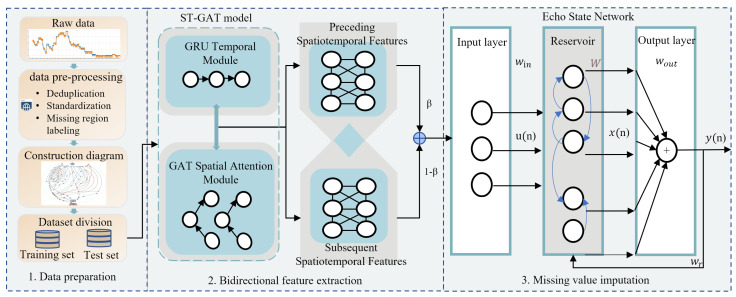
Table framework of the imputation method for missing gas time-series data based on ST-GAT-ESN.

The overall framework of the missing-data imputation method for gas time-series data based on ST-GAT-ESN is illustrated in Figure 1.

First, in the data preparation stage, the original gas-monitoring time-series data are preprocessed, including deduplication, format standardization, missing region labeling, and min-max normalization, to eliminate noise and invalid samples. Meanwhile, a spatial correlation graph is constructed according to the airflow propagation relationships among coal-mine-gas-monitoring points. The dataset is split into training and test sets in chronological order, thus providing high-quality structured inputs for subsequent feature extraction and model training.

Second, in the bidirectional spatiotemporal feature extraction stage using the ST-GAT model, the GRU temporal module is adopted to capture the evolutionary trend and dynamic dependencies of gas concentration over time. Then, the GAT spatial attention module dynamically assigns adaptive attention weights to different graph nodes according to the spatial adjacency among monitoring points, fuse the spatiotemporal correlation information of multiple sensors. The spatiotemporal features of the preceding and subsequent segments of missing regions are extracted separately, and weighted concatenation is performed to generate dual-channel fused features, which can adaptively balance the contributions of forward and backward contextual information.

Finally, in the missing-value imputation stage based on the ESN model, the concatenated dual-channel features are fed into the input layer of the ESN. The sparse connections and echo state property of the reservoir are used to capture the nonlinear dependencies embedded in the high-dimensional features. The final imputed values are mapped back to the original gas concentration range after denormalization.

### 2.2. Temporal and Spatial Feature Extraction

To address the low-imputation accuracy issue for coal mine gas time-series data caused by the loss of spatiotemporal correlation information in unidirectional feature extraction, this study adopts the ST-GAT model for bidirectional spatiotemporal feature extraction around missing regions. The proposed method captures the historical evolutionary trends before missing regions and the future constraint information after them simultaneously. The GRU temporal module is employed to characterize the dynamic concentration variations at individual monitoring points, and the graph attention mechanism is integrated to adaptively fuse the spatial propagation correlations among multiple monitoring points. This design effectively avoids the loss of contextual information caused by unidirectional feature extraction, and provides high-dimensional feature representations with complete spatiotemporal dependencies for subsequent ESN-based imputation.

#### 2.2.1. GRU Temporal Feature Extraction Module

**Figure 2:**
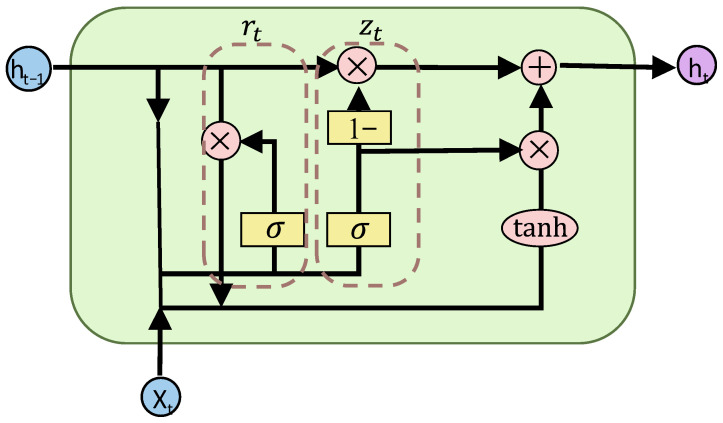
Schematic diagram of the internal GRU structure.

Recurrent neural networks (RNNs) have powerful internal memory and excellent capability in modeling sequential data, and have been widely proven effective for the learning, classification, and prediction of time-series data in practical applications. Both GRU and LSTM are variants of RNNs that can overcome the long-term dependency limitation of traditional RNNs and mitigate gradient vanishing and exploding problems in backpropagation. In comparison, the GRU has a simpler structure and higher training efficiency [25]. Therefore, considering the temporal continuity of coal-mine-gas-monitoring data, this study adopts the GRU to extract the temporal dependency features of each monitoring point, thus accurately capturing the variation trend in the gas concentration over time. The core structure of GRU is illustrated in Figure 2. It dynamically regulates the transmission of temporal information through the reset gate $r_{t}$ and update gate $z_{t}$, which can effectively alleviate the gradient-vanishing problem in long-sequence training. In this way, it effectively satisfies the requirements for long-term temporal feature extraction from coal-mine-monitoring data. The key computational formulas are given below.

(1) Reset gate controls the retention degree of the state from the previous time instant:

(1) $$r_{t}=\sigma \left(W_{r}\cdot \left[h_{t-1},x_{t}\right]+b_{r}\right)$$

In the formula, σ denotes the Sigmoid activation function, $W_{r}$ denotes the weight matrix of the reset gate, $b_{r}$ denotes the bias term of the reset gate, $h_{t-1}$ denotes the hidden state at the previous time step (*t* − 1), and $x_{t}$ denotes the input feature at the current time step (*t*).

(2) Update gate controls the update ratio of the previous state:

(2) $$z_{t}=\sigma \left(W_{z}\cdot \left[h_{t-1},x_{t}\right]+b_{z}\right)$$

In the formula, $W_{z}$ denotes the weight matrix of the update gate, and $b_{z}$ denotes the bias term of the update gate.

(3) Candidate hidden state integrates new information from current input and historical states:

(3) $${\tilde{h}}_{t}=tanh\left(W_{h}\cdot \left[r_{t}*h_{t-1},x_{t}\right]+b_{h}\right)$$

In the formula, tanh is the hyperbolic tangent activation function, $W_{h}$ is the weight matrix of the candidate hidden state, and $b_{h}$ is the bias term of the candidate hidden state.

(4) Final hidden state outputs the temporal features at the current time instant:

(4) $$h_{t}=\left(1-z_{t}\right)*h_{t-1}+z_{t}*{\tilde{h}}_{t}$$

(5) Aggregation of monitoring point temporal features: The hidden state at the last time step of the GRU is taken as the global temporal feature of a single monitoring point, and the temporal feature matrix of all monitoring points is expressed as follows:

(5) $$H_{temp}={\left[H_{temp,1},H_{temp,2},\ldots ,H_{temp,N}\right]}^{T}$$

(6) $$H_{temp,i}=h_{L}^{\left(i\right)}\left(i=1,2,\ldots ,N\right)$$

In the formula, *L* is the index of the last time step of the full-sequence temporal data of a certain monitoring point, *N* is the number of monitoring points, and $h_{L}^{\left(i\right)}$ is the hidden state of the i-th monitoring point at the last time step processed by the GRU.

#### 2.2.2. GAT Spatial Attention Module

The propagation and diffusion of gas concentration in underground coal mines follow the distribution law of the airflow field, exhibiting significant spatial correlation. The concentration variation at a single monitoring point is dynamically influenced by its upstream and downstream monitoring points. To accurately capture this spatial dependency, the GAT spatial attention module, based on the graph neural network framework, models each gas-monitoring point as a graph node and constructs an adjacency matrix according to the airflow propagation paths among monitoring points to realize the structured representation of spatial correlations. Accordingly, the spatial connectivity graph of monitoring points is defined as $G=\left(V,E,A\right)$, where *V* is the set of *N* nodes, *E* is the set of edges whose values represent the connection tightness between two monitoring points, and $A\in R^{N\times N}$ is the adjacency matrix of *V*.

The module first performs a linear transformation on the gas temporal feature $H_{temp,i}$ extracted by the GRU using Formula (7). A learnable weight matrix maps the high-dimensional temporal features to a feature space suitable for spatial attention calculation, which lays the foundation for quantifying the association strength between graph nodes.

(7) $${Wh_{i}=W\cdot H}_{temp,i}$$

To address the differences in the contribution of different monitoring points to gas concentration propagation, the self-attention mechanism is introduced to adaptively learn the spatial dependency weights among monitoring points by calculating the association score of node pair (i, j).

(8) $$e_{ij}=LeakyReLU(a^{T}\cdot \left[{Wh}_{i}|\left|{Wh}_{j}\right]\right)$$

In the formula, LeakyReLU denotes a leaky ReLU activation function, a denotes the attention vector, and || denotes the feature concatenation operation.

By combining the adjacency matrix mask, only node combinations with actual wind-flow connections are retained for computation. The associated scores are normalized using the Softmax function to obtain standardized attention weights.

(9) $$\alpha_{ij}=Softmax\left(N_{i}\right)$$

In the formula, $N_{i}$ denotes the set of adjacent nodes of node *i*.

To enhance the stability and robustness of feature extraction, the module employs a multi-head attention mechanism. K independent attention heads perform parallel calculation of spatial features, and their outputs are concatenated using Formula (10) to generate high-dimensional spatial feature representations, effectively mitigating the potential feature bias problem of single attention head architectures.

(10) $$H_{\mathrm{spat}\;}^{\left(K\right)}=\sigma \left(\sum_{j\in N_{i}}\alpha_{ij}^{k}\cdot W^{k}H_{\mathrm{temp},j}\right)$$

Ultimately, the single-head GAT output layer performs dimensionality reduction and fine-grained fusion on the high-dimensional concatenated features, generating fused features that integrate spatial correlation strength with temporal evolution patterns.

(11) $$H_{\mathrm{spat},i}=\sigma \left(\sum_{j\in N_{i}}\alpha_{ij}^{\mathrm{out}\;}\cdot W^{\mathrm{out}}H_{\mathrm{spat},i}^{\left(K\right)}\right)$$

This module eliminates the need for manual definition of spatial weights, enabling adaptive mining of dynamic correlations among gas-monitoring points and meeting the spatial feature extraction requirements in the complex underground airflow environment of coal mines.

#### 2.2.3. Missing-Value Imputation in ESN Model

**Figure 3:**
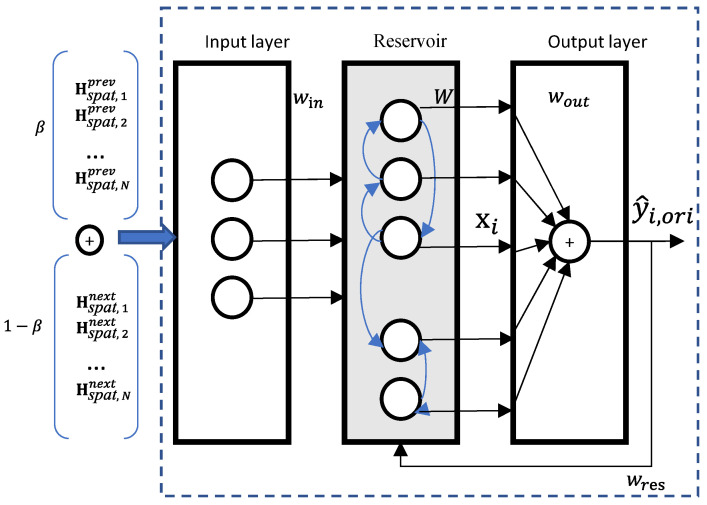
ESN structural diagram.

To address the strong contextual dependence of missing regions in coal mine gas time-series data, a dual-channel Echo State Network (ESN) is constructed to achieve accurate missing value imputation. Its core lies in fully mining the spatiotemporal correlation information before and after missing regions via dual-channel feature fusion and the echo state property of the reservoir. As illustrated in Figure 3, the ESN model consists of an input layer, a reservoir, and an output layer. Compared with recurrent neural networks, its main advantage is that it does not require iterative optimization of the sparse connection weight matrix of the reservoir. Modeling can be completed only by learning the output layer weights via ridge regression, which greatly reduces the training complexity and the risk of overfitting.

The implementation process is as follows:

(1) Dual-channel feature concatenation: For each missing region, spatiotemporal fused features of the preceding M time steps and subsequent M time steps are extracted separately. Dual-channel input features are generated through weighted concatenation by dimension.

(12) $$H_{\mathrm{dual},i}={\beta \cdot H}_{\mathrm{spat},i}^{prev}\;||(1-{\beta )\cdot H}_{\mathrm{spat},i}^{next}$$

In the formula, $H_{\mathrm{spat},i}^{prev}$ and $H_{\mathrm{spat},i}^{next}$ represent the spatiotemporal features before and after the *i*-th missing region, respectively, and *β* is the feature concatenation weight.

(2) Reservoir state update: The reservoir state is jointly determined by the dual-channel input features of the current missing region and the reservoir state at the previous moment, reflecting the core echo state property. It can accurately capture the dynamic temporal correlation of dual-channel features. The update rule is given by:

(13) $$x_{i}=\left(1-\lambda \right)x_{i-1}+\tanh\left(W_{in}H_{dual,i}+W_{res}x_{i-1}\right)$$

where *λ* is the leakage rate. $W_{in}$ is the input weight matrix, and $W_{res}$ is the sparse reservoir weight matrix after sparsification and spectral radius normalization.

(3) Output layer weight learning: The reservoir states of all missing regions are integrated into a state matrix X. Ridge regression is adopted to calculate the output layer weights. By introducing a regularization term, overfitting in small-sample scenarios is effectively avoided, and the generalization ability is improved. The calculation is expressed as:

(14) $$W_{\mathrm{out}\;}={\left(X^{T}X+\varphi I\right)}^{-1}X^{T}y$$

In the formula, X is the reservoir state matrix, y is the true label of missing values, *φ* is the regularization coefficient, and *I* is the identity matrix.

(4) Finally, the normalized preliminary imputation value is obtained by Formula (15), which is then mapped back to the real physical dimension through denormalization to output the final imputation result:

(15) $${\hat{y}}_{i,ori}=W_{\mathrm{out}}\cdot x_{i}\times \left(X_{max}-X_{min}\right)+X_{min}\;$$

In the formula, $X_{max}$ and $X_{min}$ are the maximum and minimum values of the original gas concentration data.

## 3. Experimental Results and Imputation Performance Analysis

### 3.1. Data Sources

The experimental data were collected from a coal mine in northern Linyou County, Shaanxi Province, China. According to the 2025 Mine Gas and Carbon Dioxide Emission Determination Report provided by the mine, the absolute gas emission rate is 36.38 m^3^/min, with the relative gas emission rate of 3.39 m^3^/t. Specifically, the absolute gas emission rate at the coal-mining face is 21.10 m^3^/min, and that at the heading face is 3.88 m^3^/min. The absolute carbon dioxide emission rate of the mine is 5.27 m^3^/min, and the relative carbon dioxide emission rate is 0.49 m^3^/t, which classifies the mine as a high-gas mine.

The data were acquired from the mine’s safety-monitoring system, with primary measurements collected from three gas sensors installed at the 2307 working face: the T1 gas sensor at the face head, the T0 gas sensor at the upper corner, and the T2 gas sensor in the return airway. A total of 4080 consecutive records from the above monitoring points during the continuous production period were selected as the experimental dataset.

**Figure 4:**
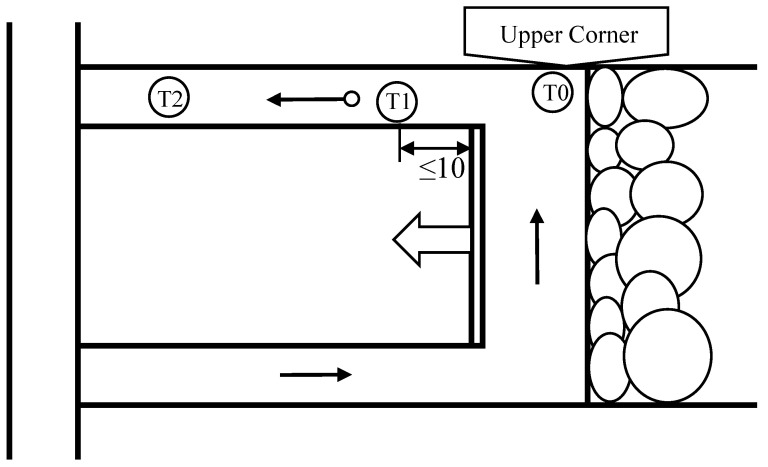
Ventilation diagram of the 2307 mining face.

The 2307 working face is located at the +820 level on the eastern wing of Panel II of the mine. The designed recoverable strike length of the working face is 2556.1 m, the inclined length is 244.6 m, the coal seam dip angle ranges from 0° to 12° (average: 3.5°), and the coal seam thickness varies from 16.1 m to 27.2 m (average: 21.55 m). This working face is characterized by complex geological structures, high gas emission, and significant nonlinear fluctuation characteristics of gas concentration. To verify the generalization performance of the proposed algorithm, experimental tests were carried out using monitoring data from sensors T0, T1, and T2 at the 1312 working face in Panel I. These two working faces adopt a classic U-type ventilation system consisting of an intake airway, as shown in Figure 4, a working face open-off cut, and a return airway, where fresh airflow enters through the intake airway and flows along the open-off cut toward the return airway. The goaf is located on the right side behind the working face; under the influence of air leakage from the goaf, desorbed gas within the goaf migrates with the leakage airflow toward the return side of the working face, forming a high-gas accumulation zone at the upper corner. Accordingly, a gas sensor T_0_ is installed here for targeted monitoring. A working-face gas sensor T_1_ is arranged in the open-off cut airflow within 10 m of the working face coal wall to real-time capture gas emission in the working space and activate linked power-off control. Meanwhile, a gas sensor T_2_ is placed in the stable airflow section of the return airway to monitor the total return gas concentration of the working face. In this manner, a three-level monitoring system covering the upper corner, working face open-off cut, and return airway is established, realizing comprehensive and hierarchical monitoring of gas emission in the U-type ventilation working face and providing critical data support for gas disaster early warning and control at the working face.

### 3.2. Experimental Design and Procedure

**Figure 5:**
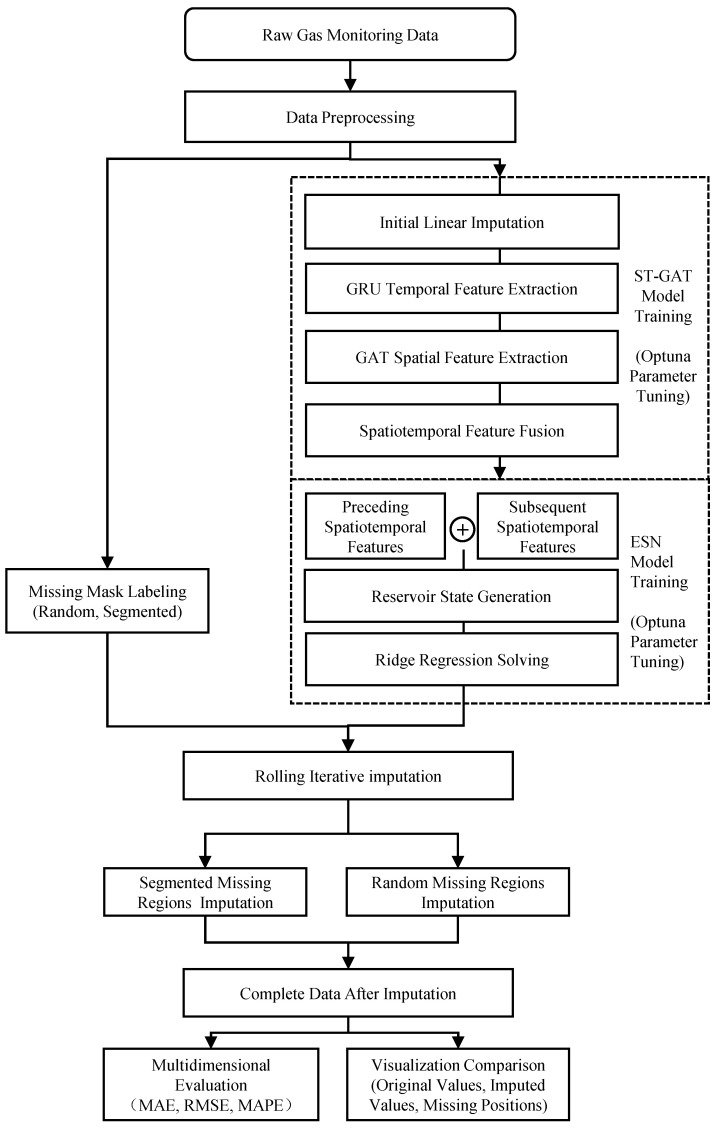
Experimental process.

In real-world production environments, methane sensors may undergo calibration or encounter power outages, malfunctions, or communication interruptions, leading to monitoring-data gaps of varying durations. To verify the generalization ability of the proposed algorithm, this study takes the 2307 and 1312 working faces as research objects, selects gas time-series data from monitoring points (T0, T1, T2) of both working faces, and designs multiple groups of controlled experiments covering two missing patterns (random missing and segmented missing) and six missing rates (5%, 10%, 20%, 30%, 40%, and 50%). Comparative experiments are conducted with various benchmark models. In addition, ablation experiments are supplemented to clarify the role and contribution of each core component of the model. The original complete data are used as the ground truth in the experiments to quantify various evaluation metrics. Considering that the randomness of random masking may lead to random deviations in single experimental results, 10 repeated tests are performed for each type of missing experiment under each missing rate to reduce the influence of random errors on the experimental conclusions. The detailed experimental procedure is illustrated in Figure 5, which is divided into five main steps:

Step 1: Data preprocessing and missing labeling. Multi-point gas time-series data from the 2307 and 1312 working faces are loaded, outliers are removed, and timestamps are aligned. A partitioning strategy combining multi-period time extrapolation and rolling validation is then adopted to separately split the data of each working face into a training set (70%), a validation set (15%), and a test set (15%). The partitioning strictly follows chronological order to avoid data leakage, and a unified splitting criterion is applied consistently without arbitrary adjustment. For the test set, mask matrices are manually constructed according to predefined missing rates to generate two missing scenarios (random missing and segmented missing) for the time-series monitoring data of T0, T1, and T2 sensors, respectively. These patterns are used to validate the generalization and robustness of the proposed model. For the training set, initial imputation is performed using linear interpolation, and a spatial correlation graph of monitoring points is constructed according to the airflow direction of the working face to generate the training input for the ST-GAT model. Notably, all statistics involved in linear interpolation and data normalization are computed exclusively based on the training set, thereby completely eliminating the risk of data leakage.

Step 2: ST-GAT training and spatiotemporal feature extraction. An ST-GAT fusion model was constructed, and hyperparameters were optimized using the Optuna tool. Following the implementation method introduced in Section 2.2, the GRU was used to extract temporal features, and the GAT was employed to learn the spatial attention weights of monitoring points, resulting in the output of spatiotemporal fused features.

Step 3: ESN training and missing value prediction. The preceding and subsequent spatiotemporal features were constructed into dual-channel windows, which were then input into the ESN reservoir to generate high-dimensional states. Ridge regression was adopted as the output layer to fit the mapping relationship, and the predicted results of missing values were output.

Step 4: Rolling iterative imputation. Imputation was performed point by point in chronological order, with real-time updates of the feature window. Random single-point and continuous segmented missing values were interpolated by category.

Step 5: Evaluation and tuning iteration. MAE, RMSE, and MAPE were selected to quantify errors, and the imputation effects were visually compared. Parameters such as the number of ST-GAT attention heads and the ESN window size were retroactively optimized until the accuracy requirements were met. The key parameter information involved is shown in Table 1. To ensure experimental fairness and rigor, parameter settings for all model components in ablation experiments were identical to the optimal parameters obtained in this step, eliminating any bias in comparative results caused by inconsistent configurations.

### 3.3. Algorithm Comparison Analysis

To verify the missing imputation performance of the proposed model for gas time-series data under multi-scenario and multi-point conditions, a quantitative analysis is conducted based on the MAE, RMSE, and MAPE metrics of different monitoring points under random missing patterns from two working faces. The results are shown in Table 2, Table 3 and Table 4. The imputation errors of the model at all monitoring points of the 2307 and 1312 working faces remain at a low level, and the overall trend is consistent with the increase in the missing rate without significant performance fluctuation, indicating that the proposed method has good generalization and robustness for different geological conditions, ventilation conditions, and monitoring positions.

Within the missing rate range of 5~30%, all errors increase slowly with the rise in missing values. When the missing rate exceeds 30%, the errors do not deteriorate sharply, reflecting that the model can still effectively mine spatiotemporal correlation features under a high missing ratio. The accuracy of the T0, T1, and T2 monitoring points in the same working face is similar, and the index differences between different working faces are slight. The standard deviations of 10 repeated experiments are all at a low magnitude, demonstrating that the results are stable and reliable and are less affected by random masks.

Based on the three evaluation metrics, the proposed ST-GAT-ESN model can effectively adapt to the complex underground monitoring environment and achieve high-precision imputation under both short- and long-term missing scenarios, which can provide stable data support for mine gas safety monitoring.

To verify the imputation performance of the model under typical engineering scenarios, such as continuous sensor failures and long-term communication interruptions, a quantitative evaluation was conducted on multiple working faces and monitoring points based on the block continuous missing pattern. As shown in Table 5, Table 6 and Table 7, compared with random missing, block missing imposes higher requirements on the ability of the imputation model to capture temporal dependencies. Nevertheless, the proposed ST-GAT-ESN model still maintains a low error level and exhibits strong fitting capability for continuous missing segments.

With an increase in the missing rate, MAE, RMSE, and MAPE exhibit overall controllable fluctuations without the sudden accuracy degradation commonly encountered under continuous missing conditions, indicating that the model can effectively compensate for feature losses caused by long-term temporal discontinuities using spatial correlation information. The error distributions at each monitoring point of the 2307 and 1312 working faces are similar, and the differences among different monitoring points within the same working face are minor. The standard deviations of 10 repeated experiments are stable, verifying the high reliability of the experimental results.

Comprehensive evaluation results demonstrate that the model possesses outstanding robustness under continuous block missing scenarios and can adapt to the complex missing types in actual mine sites. Accordingly, it can provide a more practically applicable solution for gas-monitoring data recovery in coal mines.

### 3.4. Ablation Experiments

To systematically verify the effectiveness and necessity of each core component of the proposed ST-GAT-ESN model, six groups of structured ablation comparison experiments are designed. All models are trained and tested based on the same dataset, missing pattern, missing rate range, and hyperparameter optimization strategy to ensure fair and rigorous comparisons. The ablation variants are set as follows: ① single temporal model GRU; ② single reservoir model ESN; ③ temporal feature fusion model ST-ESN; ④ spatial-reservoir fusion model GAT-ESN; ⑤ single-branch spatiotemporal fusion model single-ST-GAT-ESN; and ⑥ the complete dual-branch spatiotemporal fusion model ST-GAT-ESN in this paper.

**Figure 6:**
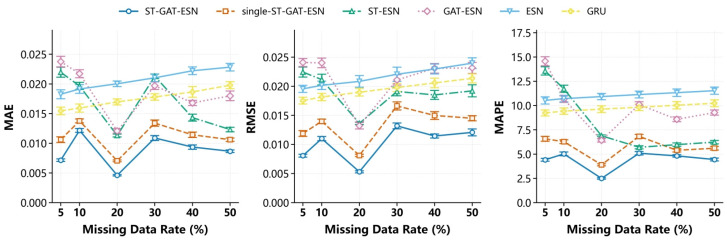
Ablation experiment results: T0 monitoring point (2307 working face) under segmented missing scenario.

**Figure 7:**
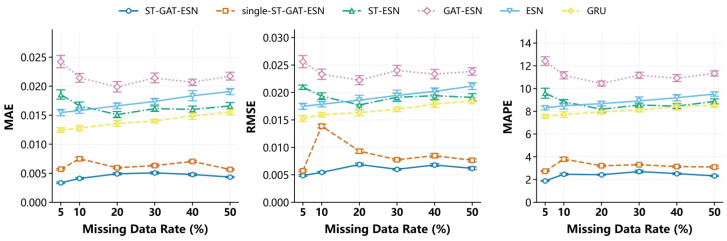
Ablation experiment results: T0 monitoring point (2307 working face) under random missing scenario.

The experimental results of MAE, RMSE, and MAPE metrics under different missing rates in the segmented missing and random missing scenarios are shown in Figure 6 and Figure 7, respectively. It can be observed that the basic single models (GRU and ESN) yield significantly high errors. Reliance on only temporal information or reservoir fitting is insufficient to characterize the complex spatiotemporal coupling characteristics of gas concentration. Although ST-ESN and GAT-ESN achieve improved accuracy, their performance is limited under high missing rates and continuous block missing scenarios due to insufficient spatial modeling and weak temporal representation, respectively. The single-ST-GAT-ESN model realizes spatiotemporal feature fusion but suffers from insufficient feature extraction constrained by its single-branch structure.

The complete ST-GAT-ESN model outperforms all other ablation variants in all metrics, and its advantages become more prominent, especially under high missing rate and continuous missing scenarios. The experiments fully demonstrate that the temporal extraction module, spatial attention module, dual-branch spatiotemporal fusion module, and ESN high-dimensional nonlinear fitting module are highly complementary, which systematically validates the rationality and superiority of the model architecture proposed in this paper.

### 3.5. Comparison and Analysis of Benchmark Models

To demonstrate the superiority of the proposed ST-GAT-ESN method, this work compares ST-GAT-ESN with several statistical imputation methods and data-driven deep learning-based imputation methods, including ARIMA, Large Gaps Data Imputation (LGDI), Multivariate Imputation by Chained Equations (MICE), Time-Series Generative Adversarial Networks (TimeGAN), Multi-directional Recurrent Neural Network (M-RNN), and Generative Adversarial Imputation Nets (GAIN) [26].

**Figure 8:**
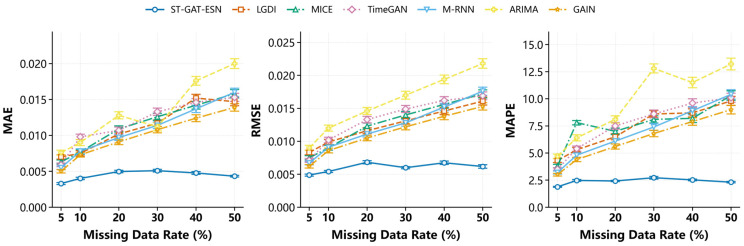
Benchmark model comparison results: T0 monitoring point (2307 working face) under segmented missing scenario.

**Figure 9:**
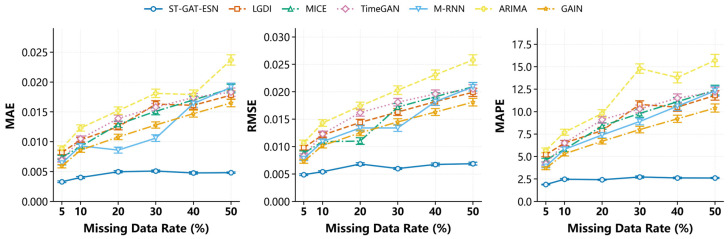
Benchmark model comparison results: T0 monitoring point (2307 working face) under random missing scenario.

The overall experimental results are presented in Figure 8 and Figure 9. In general, the ST-GAT-ESN method outperforms all other methods by a large margin. As the missing rate increases, the gradient change during training is not significant, indicating that the advantage of ST-GAT-ESN remains distinct even under high missing rates. The model can still capture spatiotemporal characteristics under such conditions, and its final convergence value is lower than those of the other six models. This implies that spatiotemporal features are preserved, and the imputed data conform to a reasonable data distribution.

The proposed method generally outperforms classical statistical methods and data-driven methods. It captures the neighborhood relationships and considers both local and global spatiotemporal correlations of the entire airflow field. Even with an increasing missing rate, ST-GAT-ESN can maintain favorable and stable imputation performance.

## 4. Conclusions

(1) To address the frequent occurrence of missing data in coal mine underground gas time-series data and the low imputation accuracy of traditional methods, the proposed ST-GAT-ESN model excavates spatiotemporal correlation features through the ST-GAT module and fits nonlinear temporal dependencies via the ESN module with dual-channel feature fusion. Compared with the contrast models, the imputation accuracy of the proposed model is improved by 30–80% under both segmented and random missing scenarios, which verifies the effectiveness of the proposed method.
(2) Experimental results demonstrate that the model maintains stable performance within the missing rate range of 5% to 50%. Especially under the high segmented missing scenario (50%), the imputation curve achieves the optimal fitting degree with the real curve, showing strong adaptability and robustness to complex missing patterns.
(3) This study provides a high-precision solution for missing-value processing of time-series data in coal mine safety monitoring. The parameter optimization logic and scenario adaptation method proposed can also provide technical reference for missing value handling in other industrial monitoring fields (e.g., environmental monitoring, equipment status monitoring). Future research can be extended to multi-source heterogeneous data fusion scenarios to further improve the generalization ability of the model.

## Figures and Tables

**Table 1:** Key parameters of the ST-GAT-ESN in this article.

Model Module	Parameter Name	Tuning Range	Optimal Value
ST-GAT timing module (GRU)	Hidden layer dimension	[32, 128]	32
	Input sequence length	[6, 24]	18
ST-GAT Space Module (GAT)	Number of attentional heads	[1, 4]	2
	Dropout rate	[0.1, 0.5]	0.3
ESN model	Window size for sequential and reverse order	[12, 48]	18
	Reservoir dimension	[128, 512]	448
	Reservoir spectral radius	[0.8, 1.0]	0.81
	Input scaling factor	[0.1, 1.0]	0.14
	Leakage rate	[0.1, 1.0]	0.56
	Regularization coefficient	[1 × 10^−8^, 1 × 10^−4^]	1.76 × 10^−8^

**Table 2:** MAE results of random missing imputation experiments for T0, T1, and T2 sensors at working faces 2307 and 1312.

Working Faces	Sensors	Missing Rates					
		5%	10%	20%	30%	40%	50%
2307	T0	0.003303 ± 0.000012	0.004010 ± 0.000011	0.004969 ± 0.000014	0.005089 ± 0.000014	0.004763 ± 0.000013	0.004315 ± 0.000011
	T1	0.003407 ± 0.000013	0.004147 ± 0.000013	0.005098 ± 0.000015	0.005228 ± 0.000014	0.004889 ± 0.000014	0.004417 ± 0.000013
	T2	0.003381 ± 0.000013	0.004095 ± 0.000014	0.005041 ± 0.000011	0.005175 ± 0.000013	0.004828 ± 0.000011	0.004384 ± 0.000011
1312	T0	0.003281 ± 0.000016	0.003981 ± 0.000018	0.004931 ± 0.000013	0.005052 ± 0.000013	0.004726 ± 0.000013	0.004286 ± 0.000013
	T1	0.003391 ± 0.000013	0.004124 ± 0.000013	0.005083 ± 0.000012	0.005197 ± 0.000015	0.004868 ± 0.000013	0.004402 ± 0.000033
	T2	0.003354 ± 0.000013	0.004068 ± 0.000013	0.005010 ± 0.000013	0.005142 ± 0.000013	0.004804 ± 0.000013	0.004359 ± 0.000012

**Table 3:** RMSE results of random missing imputation experiments for T0, T1, and T2 sensors at working faces 2307 and 1312.

Working Faces	Sensors	Missing Rates					
		5%	10%	20%	30%	40%	50%
2307	T0	0.004879 ± 0.000015	0.005423 ± 0.000014	0.006818 ± 0.000018	0.005996 ± 0.000027	0.006736 ± 0.000011	0.006177 ± 0.000016
	T1	0.004982 ± 0.000013	0.005546 ± 0.000015	0.006954 ± 0.000014	0.006121 ± 0.000007	0.006868 ± 0.000038	0.006304 ± 0.000013
	T2	0.004927 ± 0.000025	0.005481 ± 0.000025	0.006879 ± 0.000018	0.006058 ± 0.000216	0.006795 ± 0.000012	0.006237 ± 0.000011
1312	T0	0.004845 ± 0.000016	0.005382 ± 0.000014	0.006764 ± 0.000028	0.005946 ± 0.000017	0.006681 ± 0.000018	0.006127 ± 0.000014
	T1	0.004941 ± 0.000015	0.005501 ± 0.000011	0.006902 ± 0.000013	0.006075 ± 0.000014	0.006816 ± 0.000019	0.006255 ± 0.000018
	T2	0.004893 ± 0.000019	0.005437 ± 0.000015	0.006820 ± 0.000011	0.006012 ± 0.000117	0.006742 ± 0.000011	0.006189 ± 0.000019

**Table 4:** MAPE results of random missing imputation experiments for T0, T1, and T2 sensors at working faces 2307 and 1312.

Working Faces	Sensors	Missing Rates					
		5%	10%	20%	30%	40%	50%
2307	T0	1.8824 ± 0.0042	2.4665 ± 0.0053	2.4228 ± 0.0051	2.7160 ± 0.0059	2.5171 ± 0.0055	2.3127 ± 0.0020
	T1	1.9215 ± 0.0037	2.5142 ± 0.0034	2.4676 ± 0.0032	2.7675 ± 0.0062	2.5647 ± 0.0046	2.3564 ± 0.0051
	T2	1.9002 ± 0.0049	2.4893 ± 0.0025	2.4457 ± 0.0012	2.7423 ± 0.0061	2.5409 ± 0.0052	2.3348 ± 0.0055
1312	T0	1.8642 ± 0.0039	2.4431 ± 0.0055	2.4001 ± 0.0051	2.6899 ± 0.0039	2.4930 ± 0.0035	2.2907 ± 0.0060
	T1	1.9027 ± 0.0051	2.4918 ± 0.0014	2.4482 ± 0.0042	2.7450 ± 0.0050	2.5435 ± 0.0023	2.3373 ± 0.0031
	T2	1.8813 ± 0.0043	2.4669 ± 0.0053	2.4254 ± 0.0051	2.7187 ± 0.0059	2.5197 ± 0.0065	2.3152 ± 0.0043

**Table 5:** MAE results of segmented missing imputation experiments for T0, T1, and T2 sensors at working faces 2307 and 1312.

Working Faces	Sensors	Missing Rates					
		5%	10%	20%	30%	40%	50%
2307	T0	0.007264 ± 0.000021	0.012117 ± 0.000032	0.004615 ± 0.000012	0.009300 ± 0.000024	0.009300 ± 0.000024	0.008613 ± 0.000021
	T1	0.007385 ± 0.000032	0.012296 ± 0.000063	0.004689 ± 0.000004	0.009442 ± 0.000055	0.009442 ± 0.000022	0.008745 ± 0.000043
	T2	0.007321 ± 0.000022	0.012203 ± 0.000012	0.004652 ± 0.000313	0.009368 ± 0.000023	0.009368 ± 0.000045	0.008676 ± 0.000027
1312	T0	0.007192 ± 0.000021	0.011995 ± 0.000043	0.004570 ± 0.000044	0.009207 ± 0.000021	0.009207 ± 0.000023	0.008527 ± 0.000062
	T1	0.007305 ± 0.000026	0.012177 ± 0.000053	0.004643 ± 0.000017	0.009349 ± 0.000065	0.009349 ± 0.000026	0.008659 ± 0.000028
	T2	0.007243 ± 0.000021	0.012079 ± 0.000032	0.004606 ± 0.000014	0.009275 ± 0.000024	0.009275 ± 0.000024	0.008592 ± 0.000023

**Table 6:** RMSE results of segmented missing imputation experiments for T0, T1, and T2 sensors at working faces 2307 and 1312.

Working Faces	Sensors	Missing Rates					
		5%	10%	20%	30%	40%	50%
2307	T0	0.008100 ± 0.000014	0.011000 ± 0.000019	0.005304 ± 0.000016	0.011500 ± 0.000031	0.011500 ± 0.000031	0.012088 ± 0.000013
	T1	0.008225 ± 0.000025	0.011156 ± 0.000022	0.005382 ± 0.000011	0.011663 ± 0.000011	0.011663 ± 0.000134	0.012261 ± 0.000043
	T2	0.008156 ± 0.000014	0.011067 ± 0.000025	0.005340 ± 0.000036	0.011578 ± 0.000041	0.011578 ± 0.000051	0.012171 ± 0.000032
1312	T0	0.008019 ± 0.000022	0.010890 ± 0.000039	0.005251 ± 0.000026	0.011385 ± 0.000033	0.011385 ± 0.000050	0.011967 ± 0.000022
	T1	0.008139 ± 0.000024	0.011046 ± 0.000021	0.005328 ± 0.000014	0.011551 ± 0.000021	0.011551 ± 0.000091	0.012142 ± 0.000083
	T2	0.008075 ± 0.000054	0.010963 ± 0.000028	0.005287 ± 0.000011	0.011464 ± 0.000038	0.011464 ± 0.000023	0.012051 ± 0.000041

**Table 7:** MAPE results of segmented missing imputation experiments for T0, T1, and T2 sensors at working faces 2307 and 1312.

Working Faces	Sensors	Missing Rates					
		5%	10%	20%	30%	40%	50%
2307	T0	4.4340 ± 0.0102	5.0200 ± 0.0125	2.4965 ± 0.0063	4.8000 ± 0.0115	4.8000 ± 0.0155	4.4348 ± 0.0123
	T1	4.5025 ± 0.0105	5.0953 ± 0.0127	2.5340 ± 0.0023	4.8720 ± 0.0117	4.8720 ± 0.0167	4.5013 ± 0.0114
	T2	4.4664 ± 0.0103	5.0557 ± 0.0126	2.5143 ± 0.0064	4.8340 ± 0.0116	4.8340 ± 0.0109	4.4660 ± 0.0103
1312	T0	4.3897 ± 0.0101	4.9798 ± 0.0124	2.4715 ± 0.0023	4.7520 ± 0.0144	4.7520 ± 0.0114	4.3905 ± 0.0101
	T1	4.4573 ± 0.0143	5.0455 ± 0.0126	2.5091 ± 0.0064	4.8240 ± 0.0116	4.8240 ± 0.0191	4.4569 ± 0.0133
	T2	4.4210 ± 0.0102	5.0065 ± 0.0125	2.4882 ± 0.0063	4.7860 ± 0.0115	4.7860 ± 0.0105	4.4218 ± 0.0102

## Data Availability

The dataset is available on request from the authors.
